# 3D printed chitosan/polycaprolactone scaffold for lung tissue engineering: hope to be useful for COVID-19 studies

**DOI:** 10.1039/d1ra03410c

**Published:** 2021-05-28

**Authors:** Farnoush Sadat Rezaei, Ayeh Khorshidian, Farzaneh Mahmoudi Beram, Atefeh Derakhshani, Javad Esmaeili, Aboulfazl Barati

**Affiliations:** Department of Chemical Engineering, Faculty of Engineering, Amir Kabir University Tehran Iran; Department of Biology, Faculty of Basic Sciences, Gonbad Kavous University Gonbad Kavous Golestan Iran; Department of Chemistry, Faculty of Chemistry, Isfahan University Isfahan Iran; Department of Nanotechnology & Advanced Material, Materials and Energy Research Center (MERC) Karaj Iran; Department of Tissue Engineering, TISSUEHUB Co. Tehran Iran Ja_Esmaeili@yahoo.com; Department of Chemical Engineering, Faculty of Engineering, Arak University Arak Iran

## Abstract

To prevent or reduce mortality from lung diseases, new biological materials and scaffolds are needed to conduct more accurate research and support lung tissue regeneration. On the other hand, the outbreak of the COVID-19 virus and its targeting of the human lung has caused many deaths worldwide. The main aim of this study was to provide a biologically and mechanically suitable 3D printed scaffold using chitosan/polycaprolactone bioink for lung tissue engineering. Design-Expert software was employed for studying various compositions for 3D printing. The selected scaffolds underwent physiochemical, biological and mechanical studies to evaluate if they are capable of MRC-5 cell line growth, proliferation, and migration. Based on the results, the average diameter of the chitosan/polycaprolactone strands was measured at 360 μm. Chitosan concentration controlled the printability, while changes in polycaprolactone content did not affect printability. The scaffolds showed excellent potential in swelling, degradation, and mechanical behavior, although they can be modified by adjusting the polycaprolactone content. The scaffolds also revealed notable cell adhesion, nontoxicity, low apoptosis, high proliferation, and cell biocompatibility *in vitro*. To sum up, scaffold 3 (chitosan/polycaprolactone ratio: 4 : 1) revealed better activity for MRC-5 cell culture. Thereby, this scaffold can be a good candidate for lung tissue engineering and may be applicable for more studies on the COVID-19 virus.

## Abstract 2

The aim of this study was to provide a biologically and mechanically suitable 3D printed scaffold using chitosan/polycaprolactone bioink for lung tissue engineering.

## Graphical abstract

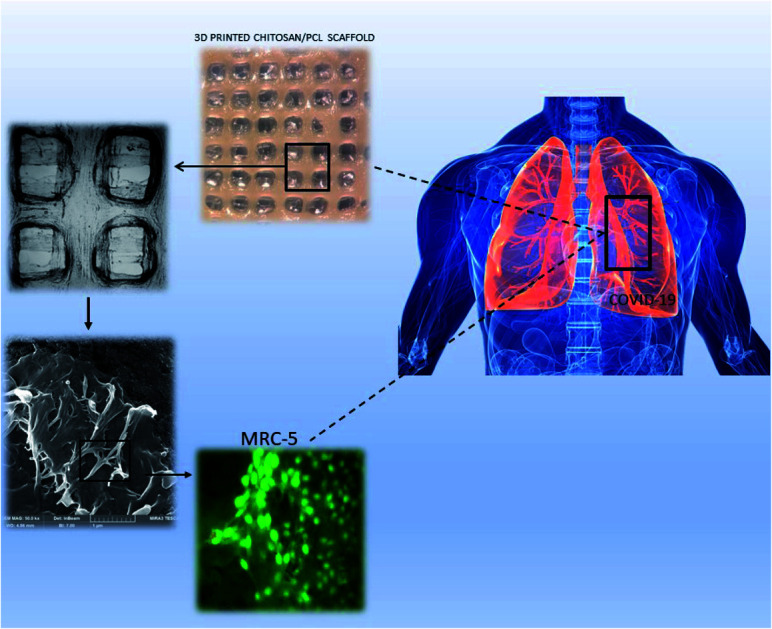

## 1. Introduction

Lung disease is known as a leading cause of malady and mortality in the world. Chronic obstructive pulmonary disease (COPD), pulmonary hypertension (PH), cystic fibrosis (CF), lung cancer, and specifically lung infection *via* coronavirus are well-known lung diseases. Currently, the only treatment with positive results is a lung transplant; however, organ transplants are hampered by the shortage of donors worldwide, the need for lifelong immune suppression, and limited success. Tissue engineering (TE) is a promising field for treating lung defects resulting from external damage or disease.^1^ Porous scaffolds are a mimic support template for tissue and cell growth.^2^ Tissue engineering and regenerative medicine using biomaterials have recently developed new technologies applicable to boost lung studies.^3^ A comprehensive review was carried out by Akter *et al.*^4^

Natural biomaterials are widely employed in TE because of their inherent bioactivity and microstructure interconnectivity, which mimics the extracellular matrix (ECM), cell adhesion, providing cell infiltration and differentiation, oxygen and nutrient transportation, and eventually retrieval of the function and structure of imperfective tissues or organs.^5^

Chitosan (CS) is biorenewable, biodegradable, biocompatible, and biofunctional, and is extensively employed in TE.^6^ The potential of CS as a biological substance is due to its cationic nature and its high charge density in solution.^7^ There are many reactive amino and carboxyl groups on CS molecules that can be chemically modified by introducing new functional groups.^8^ The *N*-acetylglucosamine moiety in CS is structurally similar to glycosaminoglycan (GAGs), which has specific interactions with growth factors, receptors, and adhesion proteins. Thus, the analogous structure in CS may also have the same bioactivity.^9^ All biopolymers have positive and negative properties in 3D printing (3DP). CS molecular weight and feed ratio affect the precision and shape of the structure. If low molecular weight CS is employed, then to neutralize the protonated amino groups, a higher concentration of sodium acetate is required.^10^ Considering the 3DP technique, reduction in the diameter of the strands results in weak mechanical properties. CS has poor mechanical properties which in the case of 3DP can be a major problem. Thereby, it is recommended to combine it with other biopolymers. Based on the targeted tissue (soft or hard tissue), to improve biological, osteoconductivity, and mechanical properties of chitosan, it is suggested to blend it with other materials such as bioglass,^11^ collagen,^12^*etc.* The desired properties for the fabricated scaffold and also the features of the targeted tissue or organ (*e.g.*, being hard or soft) need to be considered to choose the best materials.

Polycaprolactone (PCL) is a commonly used polymer in TE applications due to its strong mechanical properties and biodegradability.^13^ PCL is one of the main available biomaterials that provide a tensile strength in hydrogels and scaffolds under the crosslinking process to design appropriate constructs. So, scaffolds containing PCL have received perfect acceptance in clinical studies.^14^ There are several studies considering blends of CS and PCL for TE purposes.^15^ In research by Semnani *et al.*,^7^ a CS/PCL blend was used to fabricate an electrospun scaffold for liver TE. The made scaffold revealed good mechanical properties, hydrophilicity, cell attachment, and cell growth. In another research study, scientists proved that a CS/PCL composition can be a good candidate for TE.^16^ Considering skin TE, a CS/PCL scaffold showed its viability against HSF 1184 (human skin fibroblast cells).^17^ In the case of bone TE, a CS/PCL freeze-dried scaffold revealed its high potential in cell viability due to having homogeneous porosity and improved hydrophilic properties.

According to the previous studies, it can be concluded that the CS/PCL scaffold, depending on the CS : PCL ratio, can be used for both hard and soft tissues. Enhancement in the CS content makes the scaffold suitable for soft tissues, while increasing the PCL content makes it eligible for hard tissues. Thereby, it is hypothesized that this blend can also be nominated for lung TE. Hence, the main aim of this study was to design a new 3D printed CS/PCL scaffold capable of supporting and transporting Medical Research Council cell strain 5 (MRC-5) developed from the lung tissue for lung TE. To this aim, various CS : PCL ratios were selected according to DOE software. Creating such a scaffold would be beneficial for COVID-19-based studies regarding preclinical trials for drugs or vaccines approval.

## 2. Materials and methods

### 2.1. Materials

Chitosan (CS, Sigma-Aldrich Canada Ltd, with a molecular weight of 50 000–190 000 Da), polycaprolactone (PCL, 99%, Merck), acetic acid (100%, Merck), and chloroform (Merck) were purchased from a local supplier, TemadKala Co., Tehran, Iran. All the materials and the reagents were of analytical grade.

### 2.2. Design expert (DOE)

In this study, response surface methodology (RSM) and central composite design (CCD) were employed to find the optimum formulation to prepare the 3D printed PCL/CS scaffold with proper strand diameter, appropriate tensile strength, and best cell compatibility. For each run, the main parameters (pressure (*P*), temperature (*T*), and velocity (*V*)) were adjusted. Accordingly, the percentage of PCL and CS in the bioink composition were considered as the process parameters in DOE. Three levels, including low (−1), medium (0), and high (+1), were defined for PCL and CS concentration. For PCL, the low and high levels were 1% and 4% w/w, respectively, and for CS were 0% and 4%. According to Table 1, 13 runs were performed. The measured response was transferred in the software, which provided equations and relevant graphs to show the governing relationships between material composition and the considered response. The main aim of DOE was to find out the most optimal conditions and composition for making the scaffold.

**Table 1:** Experimental design parameters and responses

Run	Factor 1 A: chit.%	Factor 2 B: PCL%	Chit.%	PCL%	Printability
1	0	0	2	2.5	2
2	0	0	2	2.5	2
3	1	−1	4	1	3
4	−1	1	0	4	1
5	1	1	4	4	3
6	0	0	2	2.5	2
7	0	−1	2	1	1
8	−1	−1	0	1	1
9	0	0	2	2.5	2
10	−1	0	0	2.5	2
11	0	1	2	4	2
12	1	0	4	2.5	3
13	0	0	2	2.5	2

### 2.3. Bioink preparation

According to the following method, polymer solutions were prepared: first, following the DOE report, the appropriate amount of PCL was dissolved in 3 ml of chloroform. Then, an appropriate percentage of CS was weighed and added. Next, the solution was mixed to obtain a uniform solution. Finally, 5 ml of acetic acid (90% v/v) was added to the solution dropwise under sonication (170 W, 50 °C). Sonication was continued until all the CS particles were dissolved, and a yellowish viscose solution was obtained. To remove the residual chloroform, the solution was put in a desiccator under vacuum conditions. The final solution was transferred to a test tube and kept in the refrigerator until the printing process.

### 2.4. Viscosity

Viscosity of the CS/PCL solutions was determined using a RheolabQC (C-LTD180/QC) viscosimeter. The measurements were carried out at 22 °C with a shear rate of 15 s^−1^.

### 2.5. Scaffold fabrication

A 3D Bioplotter (3DPL, Iran) was employed to print scaffolds of 10 × 10 × 5 mm^3^. All groups of hydrogels were deposited using a 300 μm needle inner diameter. Magics13 EnvisionTEC software and Bioplotter RP software were used for the CAD model generation and slicing, respectively. Scaffolds were fabricated layer-by-layer. After the scaffold fabrication, 0.5 ml calcium chloride (CaCl_2_ 16%) was dispersed over the scaffold as the crosslinker. The filament width, pore sizes, and pore area of the optimum scaffold were measured using ImageJ® software. Some formulations, due to different reasons, were not printable. Each formulation possessed special conditions, including temperature (*T*), pressure (*P*), and velocity (*V*). To show the distinct behavior of the prepared bioinks, three scores have been considered: if the bioink did not work for printing (score 1), if the bioink was printable with adjusting operation parameters (score 2), easily printable (score 3). To check the fabricated scaffolds’ uniformity, at least three scaffolds were printed and evaluated in pore size and strand diameter.

### 2.6. Scanning electron microscopy

To measure the size distribution and surface structure of the 3D printed scaffolds, and also cell attachment, scanning electron microscopy (SEM) (Philips XL30; Philips, Eindhoven, Netherlands) was carried out under a 25 kV accelerated voltage after sputtering a gold layer with a 5 nm diameter on the samples. The average strand diameter was calculated using the ImageJ software (National Institutes of Health, USA).

### 2.7. Swelling test

The primary weight of the hydrogel scaffolds was measured after removing them from the crosslinker solution. The scaffolds were then incubated in 10 mM PBS solution in pH 7.4 at 37 °C and 5% carbon dioxide (according to the cell culture conditions). The samples’ weights were measured again after 1 day, 2 days, and 3 days for any mass change due to swelling. A Kimwipe was used to eliminate excess or free liquid from the scaffolds before weighing each sample. The swelling of the composite scaffolds was calculated using the following equation:1
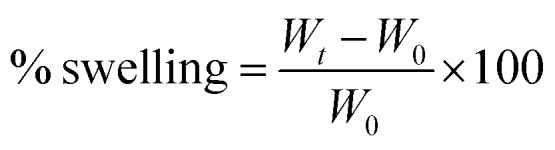
*W*_*t*_ is the hydrogel weight at the specific time, and *W*_0_ is the early weight of the scaffolds.

### 2.8. Degradation test

Scaffolds were freeze-dried and then weighed to determine their initial masses. The samples were incubated in 10 mM PBS solution in pH = 7.4 at 37 °C and 5% carbon dioxide for 3, 7, 14, and 21 days to obtain the degraded scaffolds. The PBS solution was taken out of the samples and then washed with deionized water two times, and then the samples were freeze-dried and weighed again using a digital scale. The scaffold degradation was calculated using the following equation:2
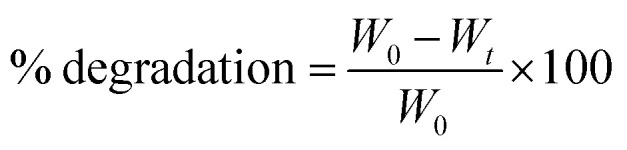
*W*_*t*_ is the freeze-dried scaffold weight at a given time, and *W*_0_ is the freeze-dried scaffold weight at the time zero.

### 2.9. Determination of porosity

The porosity of the CS–PCL scaffolds was measured by the liquid exchange method. Ethanol was chosen as the substitute liquid because it quickly penetrates the scaffold without dissolving the scaffold or changing its structure. To perform the test, the dry weight of the scaffold *W*_dry_ was measured first, and then the scaffold was immersed in ethanol for 5 minutes to obliterate air bubbles. Then the scaffold was removed and immediately placed on filter paper so that the liquid on the scaffold’s surface is absorbed in the filter paper. Finally, the weight of the scaffold *W*_wet_ was recalculated. According to eqn (3), the porosity value is calculated for each scaffold:3
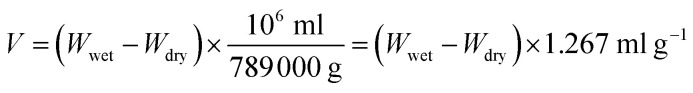


### 2.10. Tensile strength

The uniaxial tensile test was performed by a precision machine designed for this purpose. The samples remained in the same position for 20 seconds until they were firmly fixed. Then a force of about 0.02 N was applied to ensure that the specimens were attached to the clamps. Then force was applied to the samples to the point of rupture, and a stress–strain diagram was drawn for them.

### 2.11. MTT assay

First, the scaffolds were immersed in 70% ethanol for 24 hours. After drying the scaffolds at room temperature, the scaffolds (both sides) were sterilized for one hour by exposure to UV rays. The scaffolds were then placed on a plate and washed with sterile PBS. MRC-5 cells (Medical Research Council cell strain 5 is known as a diploid cell culture line which consists of fibroblasts, originally developed from the lung tissue) obtained from the cell bank in School of Advanced Technologies in Medicine (Shahid Beheshti University of Medical Sciences, Tehran, Iran) with a density of 2 × 10^5^ per milliliter were placed on scaffolds by the drip method at a rate of 20 microliters. Next, the scaffolds were incubated for 48 h at 37 °C and 5% CO_2_. At the end of the period, 10 μl of the MTT labeling reagent at the concentration 0.5 mg ml^−1^ was added to each well and incubated with them for 4 h under the same conditions (37 °C and 5% CO_2_). Then, 100 μl of the solubilization solution was added into each well. The plate was left for incubation at 37 °C and 5% CO_2_ overnight. The purple formazan crystals were checked and the absorbance was measured using an ELISA reader.^18^

### 2.12. Cell attachment study

Cell attachment of MRC-5 cells on the scaffolds was studied 48 h post-cell-seeding. The process was carried out according to a previous study.^15^ Briefly, after removing the supernatant of the scaffolds, the cell-laden scaffolds were gently rinsed using PBS (three times). Then the cultured MRC-5 cells were fixed at room temperature for 1 h using glutaraldehyde (2.5% v/v). Afterward, glutaraldehyde as a fixing solution was removed from the cell-seeded scaffolds which were rinsed slightly by PBS. The fixation process continued by sequential dehydration in various concentrations of ethanol (10%, 30%, 50%, 70%, 90%, and 100%) and air-drying overnight. The samples were then analyzed by SEM.

### 2.13. DAPI staining

In this study, 4 × 10^4^ cells were transferred to each of the sterile plates. After 24, 48 and 72 h, the cells were transferred from sterile plates to scaffolds. Then, with 1% saline phosphate buffer (PBS), they were placed in a volume of 1 ml for 10 minutes; this was repeated two times. The scaffolds were then placed in 4% paraformaldehyde solution for 10 minutes and washed again with PBS. In the next step, 0.1% Triton solution was used for 5 minutes. Then it was rewashed with PBS, and then a few drops of 4,6-diamidino-2-phenylindole dihydrochloride (DAPI, Sigma) as a nuclear stain were added to the scaffolds for 5 minutes, and again it was washed twice with PBS. It is mandatory to keep the specimens in the dark and PBS until fluorescence microscopy.

### 2.14. Live–dead assay

Acridine orange staining was performed for three scaffolds. Dual fluorescence staining solution containing 100 μg ml^−1^ acridine orange ethidium bromide (Sigma Aldrich, USA) was added to each well and washed with PBS after 5 min. The living MRC-5 cells were imaged using a fluorescence microscope (LM, Leica 090-135002, Germany).

### 2.15. Statistical analysis

Prism software and ANOVA statistical test were used to analyze the obtained results. The results were expressed as mean ± standard deviation, and *P* < 0.05 was considered a significant difference.

## 3. Result and discussion

### 3.1. Scaffold printability

Table 2 shows the viscosity and operation parameters and the results of printing scaffolds. Parameters adjustment and viscosity played a vital role in printability. Temperature, pressure, and velocity ranged between 15–25 °C, 1–2 bar, and 150–240 mm min^−1^, respectively. Amongst the prepared formulations, those which had low viscosity (L) did not show appropriate printing behavior. Scaffolds were printed by adjusting the operating parameters. Formulations 1, 2, 6, 9, and 13 as the central point (with the same formulation to detect any error in the process) showed no significant difference in viscosity (*P* > 0.05). Similar behavior was also recorded for them during the printing process. Considering the formulation numbers 4, 7, 8, 10, and 11 no significant differences were observed between the formulations of 8 & 10 (*P* > 0.05) and 4 & 7 (*P* > 0.05). Formulation 11 showed a low viscosity which failed in printing (*P* > 0.05). These formulations were not printable under any adjustments, the reason for which was attributed to the low viscosity. The formulations 3, 5, and 12 obtained the highest viscosities equal to 1000, 1340, 1270 cP respectively. Comparing to other formulations, significant differences were observed (*P* < 0.05) which illustrated acceptable behavior during printing (Table 3). Based on Table 3, by comparing the composition in different runs, it seems that CS content played the main role in printability. The optimized content of CS was considered to be 4%. Considering PCL, the PCL content (1–4%) did not affect the printability.

**Table 2:** Results for printing the prepared bioinks

Run	Printability	*P* (bar)	*T* (°C)	*V* (mm min^−1^)	Description**	Selected formula	Rheology (cP)
1	2	1	16	230	L	✗	752^a^
2	2	1	15	220	L	✗	751^a^
3	3	2	15	150	L	OK	1000^b^
4	1	—	—	—	L: not printable	✗	382^a^
5	3	1.5	25	220	H	OK	1340^c^
6	2	1	16	220	L	✗	755^a^
7	1	—	—	—	L: not printable	✗	312^d^
8	1	—	—	—	L: not printable	✗	100^e^
9	2	1	16	230	L	✗	622^a^
10	2	—	—	—	L: not printable	✗	212^e^
11	2	1	16	220	L	✗	695^a^
12	3	1	25	230	A	OK	1270^f^
13	2	1	18	240	L	✗	742^a^

**Table 3:** Selected formulations for more studies

Run	Chit.%	PCL%	Name
5	4	4	Scaffold 1
12	4	2.5	Scaffold 2
3	4	1	Scaffold 3

**Fig. 1:**
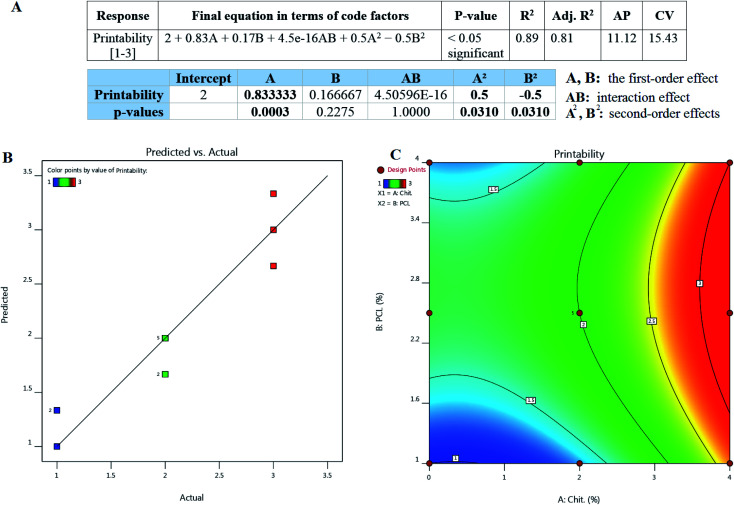
(A) The governing equation and the relevant analysis of variance results, (B) predicted *vs.* actual value plots for the printability response (ranged from 1 to 3; close to 1: not printable, and close to 3), (C) counterplot of printability: printable, red spots show each run.

A quadratic equation showing the governing relationship between the CS and PCL concentrations was proposed based on RSM studies, and printability as the response was examined by analysis of variance (ANOVA). To trust a model, the *P*-value can be a reliable index for justifying; it must be less than 0.05 to conclude that the model is valid and relevant to the experimental data. According to the RSM study (Fig. 1A), CS content (*A*) showed a significant difference (*P* < 0.0005) compared with PCL (*B*) (*P* > 0.2). As depicted in Fig. 1A, the *P*-value for the proposed equation was less than 0.05, which showed the reliability of the equation. Besides the *P*-value was also lower than 0.05 for the second-order effects (*A*^2^ and *B*^2^) and more than 0.05 for the interaction effect (*AB*). To sum up, the equation could properly predict the relationship between the parameters and responses.

The validity of the fitted model can be assessed by the determinant coefficient (*R*^2^) and its adjusted form (adj. *R*^2^).^19^ The model will be considered reliable when *R*^2^ ≥ 0.6 and has a reasonable agreement with adj. *R*^2^.^20^ As it can be seen in Fig. 1A, *R*^2^ had a value of close to 0.9 with a good agreement with adj. *R*^2^ equal to 0.81 which means that the model can be suitable for the printability of various formulations comprising CS and PCL. Adequate precision (AP) compares the range of the predicted values at the considered design points to the average prediction error, where a ratio higher than 4 is desirable. As it can be seen in Fig. 1A, AP was higher than 4 and close to 12, meaning that there was a good agreement between the recorded and predicted values of printability. The coefficient of variation (CV) shows the degree of precision with which the experiments are compared. CV equals 15 can be considered as an appropriate value for CV in reliable models in which the composition of components are the main parameters.

Fig. 1B depicts the predicted *vs.* actual plots for printability to determine the values that may be difficult to predict by the model. It signifies that the experimentally recorded values of the printability with a negligible deviation are nearly close to the values predicted *via* the optimization methodology. Fig. 1C illustrates the relationship between the printability and material composition as counterplots. By enhancing the concentration of CS, better printability was achieved. Changes in PCL did not affect the printability but it seemed that its presence improved the printability. Previous studies reported that CS still faces some limitations and needs combination with suitable biomaterials.^10^ Michailidou and colleagues reported that chitosan hydrogel showed better printability when combined with pectin.^21^ However, other studies reported that chitosan, depending on its concentration, can provide good printability with appropriate mechanical properties.^22^

### 3.2. Scaffold analysis

#### SEM and microscopic analysis

**Fig. 2:**
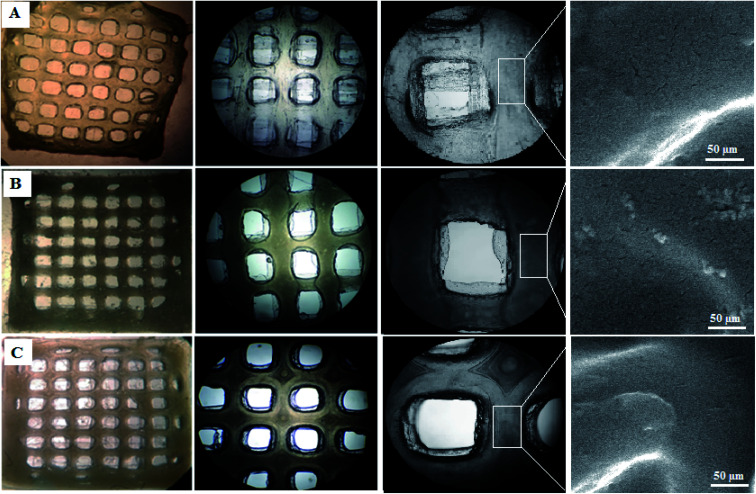
3D printing results for scaffolds. (A) scaffold 1 (contains 4% PCL and 4% CS), (B) scaffold 2 (contains 2.5% PCL and 4% CS), (C) scaffold 3 (contains 1% PCL and 4% CS).

After analysis of the printed scaffolds, according to Table 3, three scaffolds were nominated. Table 4 shows the physical parameters of the prepared scaffolds. Based on the results from the ImageJ analysis, the mean diameter of the printed strands and mean porosity were close to 360 μm and 55%, respectively. Results from the uniformity factor (UF) show that the crosslinking process increased the diameter of the strands. However, the crosslinking process had a similar effect on the scaffolds leading to UF values close to 1.2. The crosslinked scaffolds showed a smooth and uniform morphology, which means that both CS and PCL were well-dispersed in each other. In a lower magnification, all the scaffolds illustrated an organized structure (Fig. 2). It was hypothesized that this could be attributed to the Ca^2+^, which accelerates the interaction between both polymers and makes bonds between the hydrogel components.^23^ Based on the mean porosity measurements using ImageJ software, the whole porosity between the strands was close 57%. It means that the scaffolds were printed uniformly.

**Table 4:** Physical properties of the prepared scaffolds

Name	Size	Strand mean diameter	Uniformity factor (UF)	Mean porosity
Scaffold 1	10 × 10 × 5 mm^3^	357 μm	1.19	51%
Scaffold 2	10 × 10 × 5 mm^3^	365 μm	1.22	56%
Scaffold 3	10 × 10 × 5 mm^3^	359 μm	1.20	63%

### 3.3. Swelling properties, degradation, porosity, and mechanical characterization

**Fig. 3:**
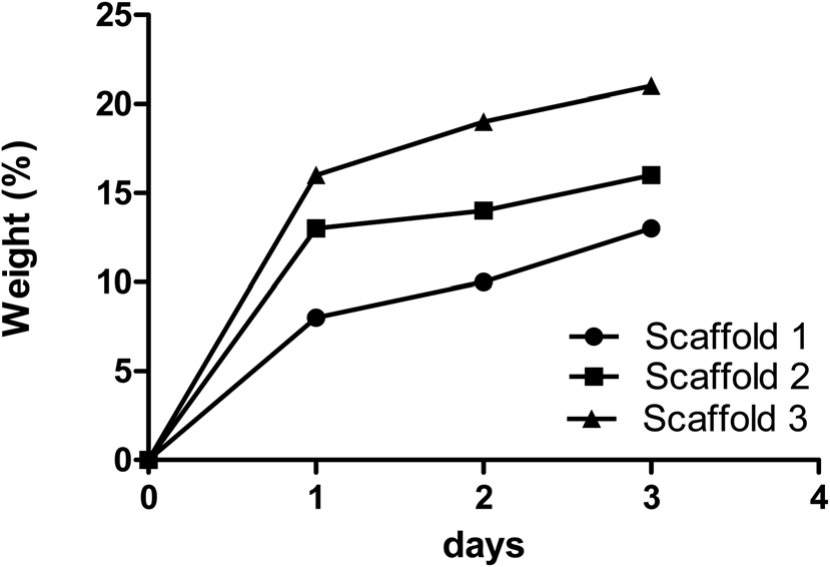
The absorption rates of the samples composed of various biomaterials are indicated by the changes in mass of the samples over time.

The swelling properties of scaffolds indicate the ability of nutrients and wastes to be exchanged between the environment and the incorporated cells in the scaffold to produce synthetic tissue. The swelling efficiency directly refers to the hydration ability and stability inside the biological systems.^24,25^ All samples in this study were incubated in PBS to assess the rate of water absorption over time. The change in the scaffolds’ mass due to water absorption showed an alike trend, as shown in Fig. 3. Scaffolds 1, 2, and 3 showed 13%, 16%, and 21% swelling, respectively, after 72 h of incubation. According to the data, scaffold 3 revealed the highest absorption rate compared with other samples. The reason can be attributed to its low PCL content.^26^ Scaffold 1, due to its high PCL content, revealed the lowest swelling potential during incubation. It has been reported that the swelling potential of the scaffolds can be affected by the degree of crosslinking, amorphous regions, and level of hydroxyl groups.^27–29^ Comparing the three scaffolds, they had the same CS content but were different in PCL content. It could be concluded that PCL concentration directs the potential swelling properties in the printed scaffolds.^30^

**Fig. 4:**
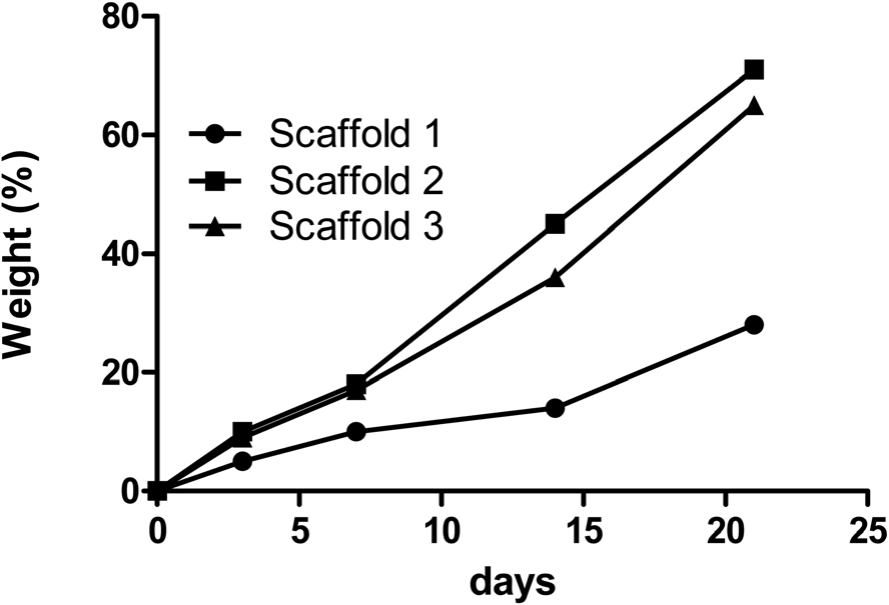
The rates of degradation of the samples composed of PCL/CS are indicated by the changes in the samples’ mass over time.

The degradation rate of each scaffold was also measured by observing the change in the samples’ mass after immersion in PBS over time. Fig. 4 depicts the degradation behavior of the scaffolds during incubation. 28%, 65%, and 71.5% degradation and same patterns were reported for scaffolds 1, 2, and 3, respectively, over time incubated in PBS. Scaffold 1 showed a slow degradation process compared with the other scaffolds from the beginning. During the first week, scaffolds 2 and 3 had almost the same degradability, but from the seventh day onwards, scaffold 3 had more degradability. Various reasons can influence degradation behavior. Ki-Taek Lim *et al.*^31^ reported that crosslinker and the time of crosslinking could affect the degradation process. In this study, it was tried to do the same process for crosslinking to control the role of crosslinking during degradation. By the way, the changes in the concentration of the components may lead to a different level of crosslinking.^32^ Basically, PCL has slower degradation compared with CS.^33^ As well as swelling analysis, due to the same content of CS in all scaffolds, it was hypothesized that the PCL concentration can be considered as the effective parameter in degradation behavior. The higher PCL content resulted in higher crosslinking and limiting degradation.^34^

**Fig. 5:**
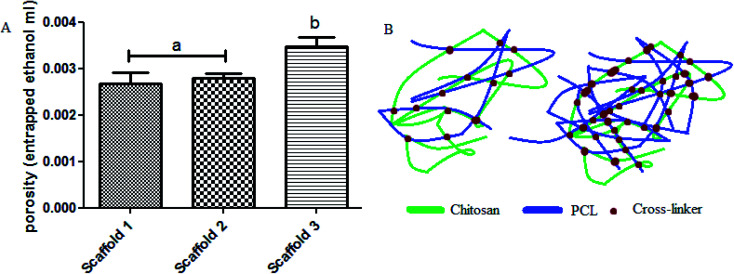
(A) The porosity results for the scaffolds, (B) schematic of polymer chains entanglement showing the level of porosity.

Considering the porosity within the scaffolds’ structure (especially strands), measuring the porosity has been carried out by liquid (ethanol) displacement. Results obtained using eqn (3) are shown in Fig. 5. Based on Fig. 5A, the porosity in scaffold 1 was 0.0027, scaffold 2 was 0.0028, and in scaffold 3 was 0.0035. Scaffold 3 showed a significant difference compared with the other scaffolds (*P* < 0.05). There was no significant difference between scaffold 1 and scaffold 2 (*P* > 0.05). Porosity is known as a vital parameter in biological scaffolds. Scaffolds need high interconnected porosity to provide a good and uniform flow of nutrients for cell growth.^35^ Scaffold 3, compared with other scaffolds, possessed less PCL, which means that the entanglement between the polymer chains is notably lower, thereby after crosslinking, huge and more connected pores will be obtained (Fig. 5B).

**Fig. 6:**
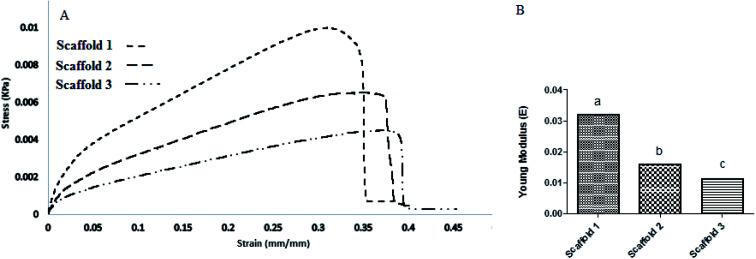
Stress–strain diagram (a) scaffold 1, (b) scaffold 2, and (c) scaffold 3.

The mechanical behavior is a vital factor in deciding about the quality of a scaffold. The tensile strength (MPa) of all samples was determined by finding the elastic modulus (EM) of each sample, and the results are shown as strain–stress curves in Fig. 6A. Scaffold 1 revealed a significant difference (*P* > 0.05) in the viewpoint of EM compared with scaffolds 2 and 3. Scaffold 3 showed the lowest EM (Fig. 6B). PCL is known as a polymer that improves the mechanical behavior of scaffolds in TE.^36^ It seems that the PCL content can be the main factor affecting the value of EM. The CS : PCL ratio in scaffold 1 was 1 : 1, while for scaffold 2 and 3 was 2 : 1 and 4 : 1, respectively. Thereby, it can be concluded that PCL controls the EM. As it can be seen, the elastic zone in scaffolds 2 and 3 was longer than in scaffold 1, which means that under small energy, they show more elongation, which is hypothesized to be suitable for lung expansion mimicking. This behavior may be useful in lung TE. One of the main properties of the respiratory system is its ease of expansion and contraction in respiration.^37^ Comparing scaffolds 2 and 3, scaffold 3 due to lower PCL revealed better elastic behavior (Fig. 6A). Pinelopi Andrikakou and colleagues carried out research about the behavior of lung tissues of rabbits and rats under tension.^38^ It was interesting when our results were compared with their findings. They reported that tissues from both animals revealed similar behavior under tension but generally, rat tissue showed higher values of stress. This distinct behavior can be considered because of differences in composition. The results from the elongation behavior of rabbit lung tissue were close to that of scaffold 3. In our study, the stress of 0.0042 kPa resulted in a strain of 0.39, while they reported a strain of 0.4 for the stress of 0.004 kPa (for rabbit) and a strain of 0.4 for the stress of 0.0037 kPa. It can be hypothesized that scaffold 3 could be nominated for lung *in vitro* studies as an alternative.

### 3.4. Biocompatibility (cytotoxicity, apoptosis, cell adhesion and morphology)

**Fig. 7:**
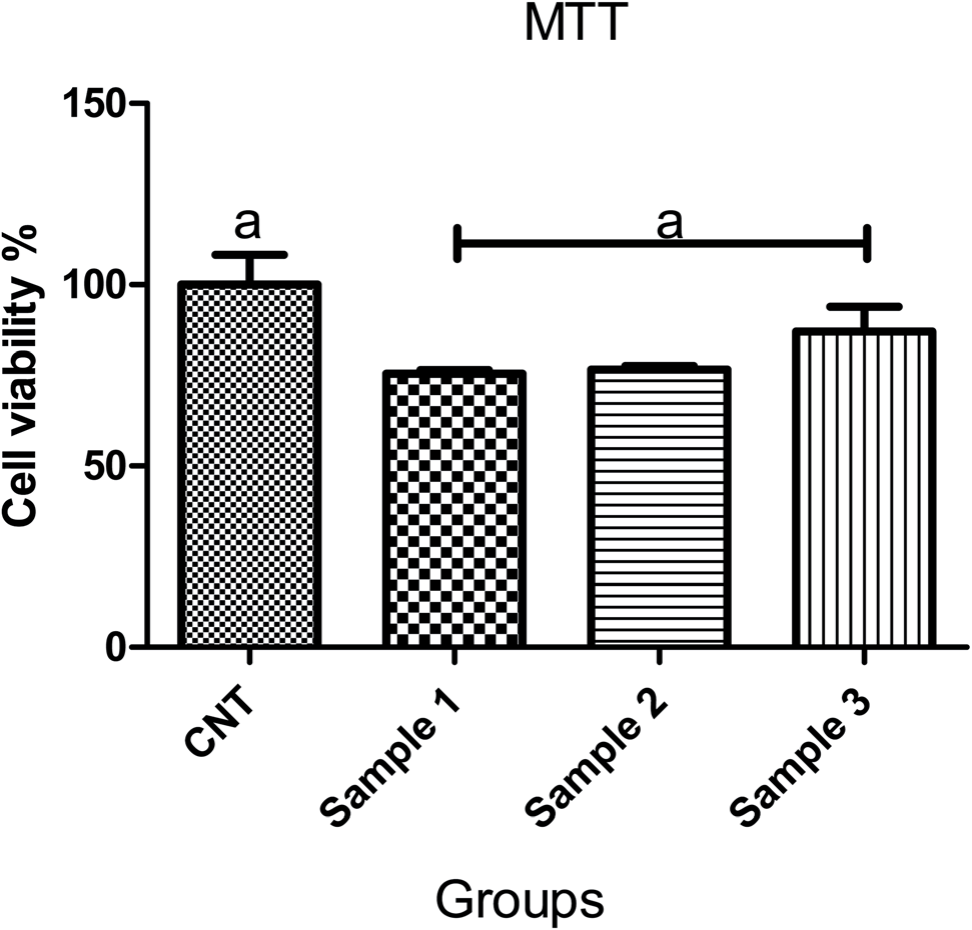
Comparison of cell viability on 3 samples (*P* < 0.05).

The scaffolds are supposed to be employed in TE; thereby, it is necessary to assess their cytotoxicity and biocompatibility. The MTT test assessed the suitability of scaffolds for MRC-5 cells viability, as shown in Fig. 7. According to the cytotoxic assay, there was no significant difference (*P* > 0.05) between the scaffolds and the control group, which means that all three scaffolds are suitable for cell culture. Comparing the scaffolds with each other, scaffold 3 revealed good efficacy in cell viability, which indicated that cell proliferation was enhanced. Based on the results from swelling and porosity assessments, this difference may be attributed to the higher porosity and low PCL content. On the other hand, based on the cell growth and also cell concentration in each scaffold, it can be claimed that scaffold 3 revealed good cell adhesion compared with the other scaffolds. Enhancement in the PCL content can affect cell adhesion. Similar results covering the good proliferation and adhesion of cells in CS-based scaffolds have been reported in previous studies.^39–41^ In a research study it has been shown that CS has the potential for bone tissue engineering when combined with polymeric or ceramic materials such as hydroxyapatite.^42^ The scientists revealed CS biocompatibility against a variety of cell lines including osteoclast,^43^ fibroblast,^44^ chondrocytes,^45^ and so on (nicely reviewed by Kim^46^).

**Fig. 8:**
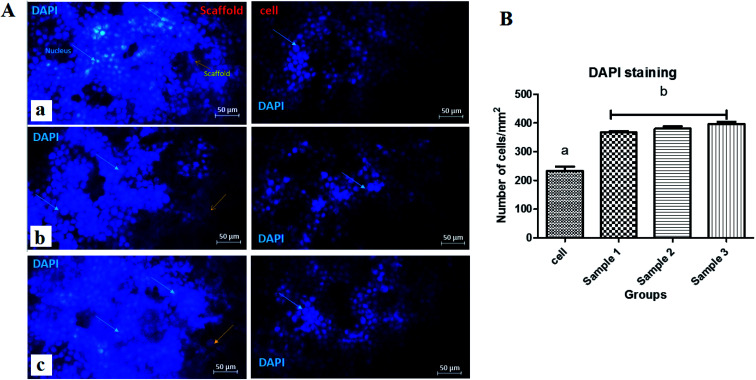
(A) Fluorescence micrographs of the DAPI stained MRC-5 cells on (a) scaffold 1, (b) scaffold 2, and (c) scaffold 3 (scale bar: 50 μm), (B) DAPI staining diagram for the scaffolds indicating the concentrations of the MRC-5 cells.

**Fig. 9:**
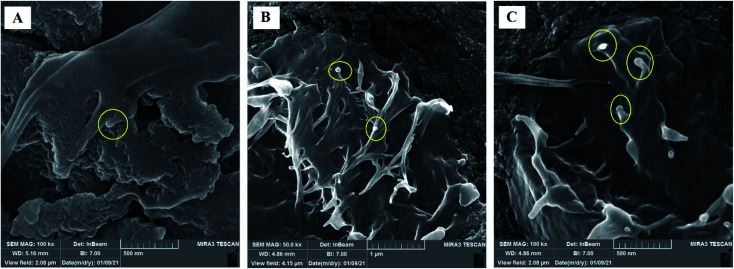
SEM images of MRC-5 cell-laden scaffolds showing cell attachment and their spread on the surface of (A) scaffold 1, (B) scaffold 2, (C) scaffold 3.

Staining is used to check the number of living cells on the scaffold. DAPI stands for diamino-phenyl indole, which is a fluorescent dye used for DNA staining.^47^ One of the purposes of this type of staining is to study the cell cycle, determine the index of mitosis in an organism, or count cells and bacteria. After staining the cells with DAPI, the cells were imaged using a fluorescence microscope. As the results of DAPI staining, the cell nuclei were observed under a fluorescence microscope as shown in Fig. 8A. In the microscopic observations, the cell nuclei were visible both on the surfaces and inside the scaffold cavities. Cells seemed to be attached to the scaffolds and also revealed higher concentrations. According to Fig. 8B, there was a significant difference between all scaffolds compared to the control group (*P* < 0.01). No significant difference was reported amongst the scaffolds, but scaffold 3 revealed better results than scaffolds 1 and 2. Adhesion of cells to their ECM is one of the crucial properties for *in vitro* differentiation and proliferation.^48^ Choosing the appropriate material for scaffolding and obtaining high adhesion of cells is of concern in this regard. SEM images of the cell adhesion on the scaffolds are depicted in Fig. 9. As it can be seen, the cells attached and started spreading over the scaffold’s surface over time. The images illustrate that cells grew and proliferated on the surface of all scaffolds. Most of them depicted several extensions, while normal spherical shapes were also observed (marked by a yellow circle). Also, the scaffold had enough porosity to provide a microenvironment and free migration. This attachment shows the surfaces of the scaffolds were adequately hydrophilic. It seems that the scaffold’s surface possesses enough roughness, which is a vital parameter in improving cell attachment.^49^ Similar results were reported previously by Tao Lou *et al.*,^50^ confirming the potential of CS/PCL in HaCat and hFF cell attachment. In another study, Mirzaei *et al.* showed that the CS/PCL composition has a good potential in cell attachment.^15^ They also reported that this composition can provide a suitable surface roughness. Besides, based on the DAPI staining results, the MRC-5 cells improved the cell-attachment potential of the scaffold’s surface after 48 h in a suitable concentration (Fig. 8). Also, the cell morphology was confirmed by the results of the DAPI staining. After 48 h of cell culture, the MRC-5 cell seemed healthy without any changes in morphology. These results proved that the formulated bioinks made of CS and PCL are compatible with the MRC-5. It must be noted that the lower content of PCL led to better results. There are reports regarding CS/PCL-based scaffolds for TE purposes. For instance, Naznin Sultana *et al.*^17^ used a PCL/CS composition to prepare freeze-dried scaffolds for TE. They showed that this scaffold possesses the ability to control bacterial infections. It was reported that CS enhanced wettability and permeability, accelerated PCL hydrolytic degradation, and improved PCL cell recognition sites.^51^ CS’s main functional groups, such as hydroxyls, acetamides, and amines that are very reactive, are the main reason for its unique abilities in TE.^52^

**Fig. 10:**
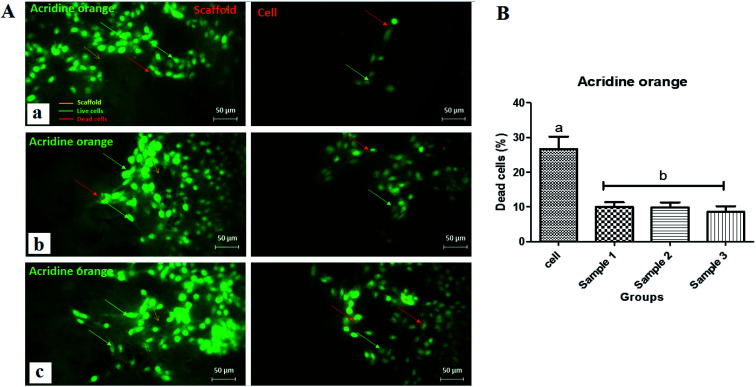
(A) Fluorescence micrographs of the acridine orange stained MRC-5 cells on (a) scaffold 1, (b) scaffold 2, and (c) scaffold 3 (scale bar: 50 μm), (B) acridine orange staining diagram for scaffolds showing the level of the dead cells.

Live–dead assays were carried out using acridine orange staining as shown in Fig. 10. Interestingly, the results revealed that the concentration of dead cells was low for all scaffolds indicating the nontoxicity of the scaffolds and increased cell growth and proliferation on scaffolds. After AO/ethidium bromide (EtBr) staining, fluorescence microscopy allows visualization of apoptosis-related changes in MRC-5 cells and their nuclei to verify cell viability. Live cells appear green (with acridine orange), while apoptotic cells appear orange (with EtBr). Initially, MRC-5 cell adhesion and spreading over the 3 scaffolds would be considered an essential step for cell growth. All images (Fig. 10A) proved that the normal morphology of spindle-like shapes of live MRC-5 cells was maintained in the 3 samples. Fluorescence images also proved that the adhering MRC-5 cells are viable, represented by the lysosomes stained by AO, and suggest the absence of dead cells since the presence of EtBr positive cells has not been observed. There was no significant difference between the 3 scaffolds compared with the control group (Fig. 10B). However, comparing the scaffolds, the highest percentage of cell viability, cell growth and proliferation, and the least dead cells were reported for scaffold 3. Therefore, it was hypothesized that the fabricated hydrogel scaffolds are more responsive to surface cell seeding than cell-laden culture. The surface of the CaCl_2_-crosslinked CS/PCL scaffolds was nearly smooth without any crack or aggregations, which can lead to the hypothesis that it may mimic the fine microenvironment of the natural ECM and facilitated protein adsorption onto the surface of the printed scaffolds. Furthermore, the addition of Ca^2+^ and strong interaction between CS and PCL results in a highly homogeneous structure suitable for cell adhesion and cell-to-cell communication.^53,54^

## 4. Lung TE and COVID-19

A novel coronavirus (2019-nCoV) outbreak in late December 2019 has led to a global pandemic known as novel coronavirus disease (COVID-19). TE as a new major technique that appeared in the 21st century has revealed its high potential in fascinating research and new therapeutics. Many scientists over the past year altered their research to focus on COVID-19. Hospitals have canceled their non-emergency activities and clinical trials to invest their time and activities on COVID-19-based studies.

During the COVID-19 epidemic, the expansion of reliable 3D constructs with TE has helped as a platform for the validation of antiviral therapies, studying the mechanism of the COVID-19 virus.^55^ Currently studying cultured cell lines, animal models, primary tissue-derived cells, human organoids, and *in vitro* lung tissue cultures show infection by respiratory viruses; nevertheless, all of these preclinical models have significant limitations. Surely, conventional media fail to form specific tissue constructs as well as living human organs. Researchers’ initial efforts in 3D studies using explants of human respiratory tract tissue culture bypassed the limitations associated with a 2D cell or Petri dish culture.^56^ However, the donor availability is limited, and also it is not possible to do long-term cultures; thereby, it seems that the explant cultures are limited.^57^

COVID-19 is a dangerous disease caused by the new coronavirus, prompting lung difficulties such as pneumonia and acute respiratory distress syndrome (ARDS) in several severe cases. Sepsis, another possible problem of COVID-19, can also prompt lasting infliction on the lungs and other organs.^58^

In pneumonia, the lungs become filled with fluid and inflamed, leading to breathing troubles. For some people, breathing problems can become difficult enough to need treatment at a hospital with oxygen or even a ventilator. Pneumonia that COVID-19 causes tends to take hold in both lungs.^59^ The air sacs, one of the main parts of the lungs, are filled with fluid from the infection, and their oxygen capacity is limited, causing coughing and shortness of breath. Most people survive pneumonia without lasting lung damage, while pneumonia connected with COVID-19 may be severe. Even after getting rid of COVID-19, lung injury may result in breathing troubles that might take months to recover.^60^ ARDS patients mostly cannot breathe independently and need ventilator support to help oxygen circulation in their body. People who have recovered from ARDS and survived COVID-19 likely have lasting pulmonary scarring.^61^ Another possible complexity of a severe case of COVID-19 is sepsis. Sepsis occurs when an infection reaches and spreads through the bloodstream, causing tissue damage everywhere it goes.^62^ Lungs, heart, and other body systems are always working together, but the interaction between the organs falls apart in sepsis. Other organ systems can begin to stop working, one after another, such as the lungs and heart. Even when resolved, sepsis can damage the patient’s lungs and other organs forever.^63^

The recommended 3D printed CS/PCL scaffold inherits enough porosity to simulate lung structure. Fluids and oxygen could penetrate through the scaffold. This scaffold also showed its potential in MRC-5 cell attachment, growth, and proliferation as representative of lung tissue (biological studies). Instead of using 2D cell culture, animal study, or biopsy, this scaffold can be a good candidate for more studies. Infecting the cell-seeded CS/PCL scaffold with the COVID-19 virus can let us test more drugs and vaccines. For more accurate results, integrating this scaffold with microfluidic devices will be beneficial to study ARDS or sepsis or other side effects.^55^

## 5. Conclusion and future prospects

CS/PCL scaffolds with various compositions based on the DOE study were successfully fabricated through the 3D printing method. 3D bioprinting, as a novel technique to fabricate scaffolds, helped to accurately construct scaffolds in the desired forms. In the case of lung TE, this technique is hypothesized to succeed in fabrication of the scaffold with the desired porosity in any shape. In this study, this technique made it easier to create a structure to evaluate the viability of the MRC-5 cells in the presence of different ratios of CS : PCL. 3D bioprinting could arrange the strands in a desired distance and manner (as a small part of the main scaffold in the shape of human lung) to optimize cell attachment, proliferation, and migration to boast regeneration processes.

The main aim of this study was to produce a CS/PCL scaffold to assess the growth of MRC-5 cells for more lung studies, which can be a promising microenvironment for COVID-19 studies. In this regard, it was tried to evaluate the mechanical properties and also cellular compatibility. The three optimized scaffolds were nominated for evaluation. Based on the outcomes, a combination of CS and PCL could cause notable mechanical integrity for the structures similar to that of rabbits and rats, in addition to providing a suitable microenvironment for cell growth. The presence of CS in the main structure was known as one of the main reasons for improving the biological properties of the scaffolds by influencing the swelling ratio, surface wettability, and weight loss percentage of PCL. The biological studies revealed that cells could grow, proliferate and migrate within the scaffolds. To sum up, amongst the nominated scaffolds, scaffold 3, with a CS : PCL ratio of 4 : 1, due to lower PCL content showed higher potential in lung TE; thereby keeping coronavirus issues in mind, this scaffold can be recommended for more research to study the behavior and effect of COVID-19 virus in lung tissue.

The main challenge in lung TE is the expansion and contraction (inhalation and exhalation) of lung tissue, where the cells are constantly under stress, and we need to assess this as well. Trapped oxygen in the lungs can also be another challenge. Maintaining these applications during scaffolding degradation is also an important challenge, so that scaffold alignment should not affect cell behavior during the reconstruction process. The rest of our project focuses on alveolar type I cells (AEC I) and cuboidal alveolar type II cells (AEC II) culture on the recommended scaffold and manufacturing a microfluidic system where the scaffold experiences oxygen inhalation and exhalation during cell culture.

## Conflicts of interest

There are no conflicts to declare.

## Supplementary Material
